# RhoA rescues cardiac senescence by regulating Parkin-mediated mitophagy

**DOI:** 10.1016/j.jbc.2023.102993

**Published:** 2023-02-08

**Authors:** Joanne Ern Chi Soh, Akio Shimizu, Md Rasel Molla, Dimitar P. Zankov, Le Kim Chi Nguyen, Mahbubur Rahman Khan, Wondwossen Wale Tesega, Si Chen, Misa Tojo, Yoshito Ito, Akira Sato, Masahito Hitosugi, Shigeru Miyagawa, Hisakazu Ogita

**Affiliations:** 1 Division of Molecular Medical Biochemistry, Department of Biochemistry and Molecular Biology, Shiga University of Medical Science, Otsu, Japan; 2 Department of Emergency, The Fourth Affiliated Hospital of China Medical University, Shenyang, China; 3 Division of Legal Medicine, Department of Social Medicine, Shiga University of Medical Science, Otsu, Japan; 4 Department of Cardiovascular Surgery, Osaka University Graduate School of Medicine, Suita, Japan

**Keywords:** cardiomyocyte, cardiomyopathy, mitophagy, Parkin, Ras homolog gene family, member A (RhoA), senescence, signal transduction, AAV, Adeno-associated virus, ANOVA, Analysis of variance, BP, Blood pressure, BSA, Bovine serum albumin, CCCP, Carbonyl cyanide m-chlorophenyl hydrazone, cKO, Conditional knockout, DCM, Dilated cardiomyopathy, FFPE, Formalin-fixed paraffin-embedded, H-E, Hematoxylin and Eosin, HR, Heart rate, HRP, Horseradish peroxidase, LV, Left ventricular, LVAD, Left ventricular assist device, LVDd, Left ventricular end-diastolic diameter, LVEF, Left ventricular ejection fraction, LVPWd, Left ventricular posterior wall thickness at end diastole, PAGE, SDS-polyacrylamide gel electrophoresis, PBS, Phosphate-buffered saline, PD, Parkinson’s disease, PINK1, PTEN-induced putative kinase 1, PMSF, Phenylmethylsulfonyl fluoride, RIPA, Radioimmunoprecipitation assay, ROCK, Rho kinase, SAβ-gal, Senescence-associated β-galactosidase, TEM, Transmission electron microscopy, Ub, Ubiquitin

## Abstract

Heart failure is one of the leading causes of death worldwide. RhoA, a small GTPase, governs actin dynamics in various tissue and cell types, including cardiomyocytes; however, its involvement in cardiac function has not been fully elucidated. Here, we generated cardiomyocyte-specific RhoA conditional knockout (cKO) mice, which demonstrated a significantly shorter lifespan with left ventricular dilation and severely impaired ejection fraction. We found that the cardiac tissues of the cKO mice exhibited structural disorganization with fibrosis and also exhibited enhanced senescence compared with control mice. In addition, we show that cardiomyocyte mitochondria were structurally abnormal in the aged cKO hearts. Clearance of damaged mitochondria by mitophagy was remarkably inhibited in both cKO cardiomyocytes and RhoA-knockdown HL-1 cultured cardiomyocytes. In RhoA-depleted cardiomyocytes, we reveal that the expression of Parkin, an E3 ubiquitin ligase that plays a crucial role in mitophagy, was reduced, and expression of N-Myc, a negative regulator of Parkin, was increased. We further reveal that the RhoA–Rho kinase axis induced N-Myc phosphorylation, which led to N-Myc degradation and Parkin upregulation. Re-expression of Parkin in RhoA-depleted cardiomyocytes restored mitophagy, reduced mitochondrial damage, attenuated cardiomyocyte senescence, and rescued cardiac function both *in vitro* and *in vivo*. Finally, we found that patients with idiopathic dilated cardiomyopathy without causal mutations for dilated cardiomyopathy showed reduced cardiac expression of RhoA and Parkin. These results suggest that RhoA promotes Parkin-mediated mitophagy as an indispensable mechanism contributing to cardioprotection in the aging heart.

Heart failure is a multifaceted disease with a complex etiology. It remains a major public health problem and is the leading cause of death worldwide, with high morbidity and mortality rates (1, 2, 3, 4). Heart failure is a chronic pathophysiological state in which the heart muscle is unable to pump an adequate supply of blood to the whole body due to progressive loss of myocardial contractile function over time (5). Despite medical advances, the prognosis of patients with heart failure remains poor (6), and current therapeutic approaches seem palliative as the underlying mechanisms contributing to heart failure are still not fully addressed.

The heart is a highly metabolic organ in which mitochondrial dynamics are precisely regulated to ensure optimal mitochondrial function (7, 8). Given the high energetic demand of the heart, age-related defects in mitochondrial bioenergetics can have detrimental effects on normal cardiac pumping. Accumulation of dysfunctional mitochondria is associated with suppression of mitophagy (9, 10), leading to a defect in mitochondrial quality control. Mitophagy is an evolutionarily conserved mechanism that plays a crucial role in the mitochondrial quality control (11). It enables the degradation of damaged and superfluous mitochondria in response to cardiac stress, including senescence.

RhoA is a small GTPase that regulates diverse cellular events, including actin cytoskeleton organization, cell adhesion, migration, invasion, apoptosis, extracellular matrix remodeling, and smooth muscle contractility (12, 13). RhoA is ubiquitously expressed in almost all tissue and cell types, including cardiomyocytes. RhoA signaling plays a pivotal role in processes leading to cardiovascular diseases, such as pulmonary hypertension, vasospastic angina, and heart failure (14, 15). Thus, RhoA function in the heart remains an interesting focus among molecular cardiologists as well as biologists. However, the understanding of the molecular signaling of RhoA in the heart is still incomplete.

In this study, we found that cardiomyocyte-specific RhoA conditional knockout (cKO) mice had a significantly shorter lifespan with features of early senescence, severely impaired cardiac function, and build-up of many structurally disorganized and enlarged mitochondria compared with control mice. These phenotypes suggest a causative link between cardiac aging and mitochondrial dysfunction with regard to RhoA signaling. We further revealed the molecular mechanisms of cardiac RhoA in regulating mitochondrial dynamics, which may protect the heart from senescence-mediated dysfunction.

## Results

### Deterioration of cardiac function and early death in RhoA cKO mice

**Figure 1:**
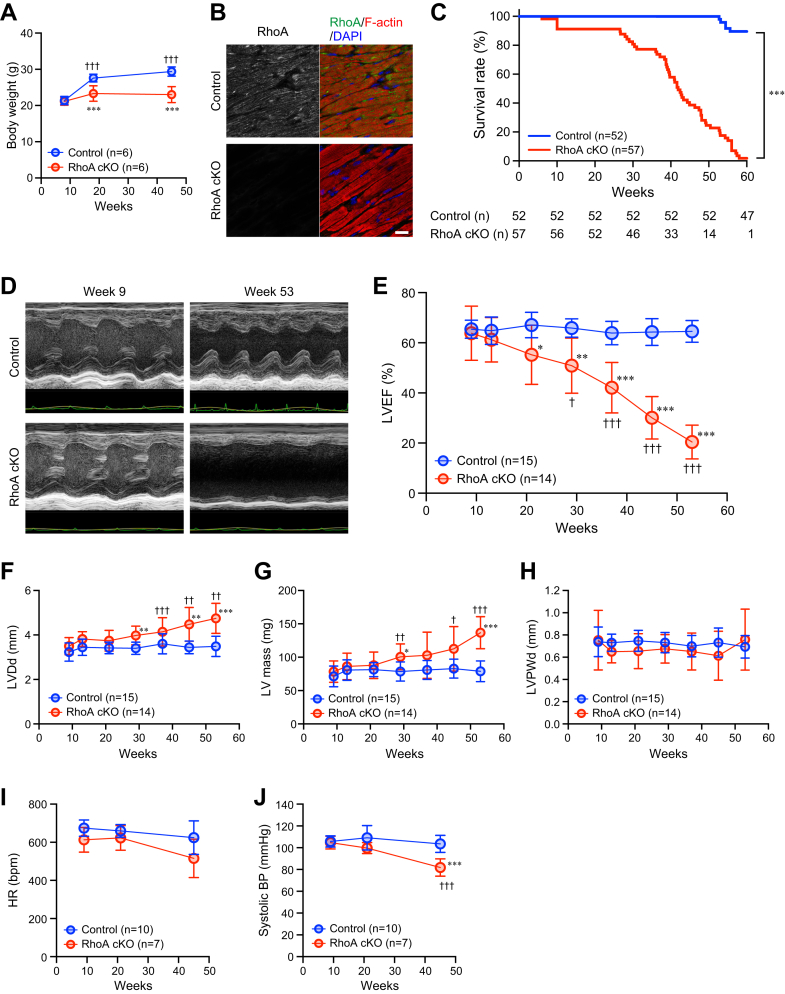
**Shorter lifespan and impaired cardiac function in RhoA cKO mice.***A*, body weight of 9-, 18-, and 45-week-old mice. *B*, immunostaining for RhoA in the heart at 9 weeks after birth. F-actin and nuclei were counterstained with phalloidin and DAPI, respectively. Scale bar: 20 μm. *C*, Kaplan–Meier survival curve of mice. The number of mice (n) in each group at every 10 weeks is indicated below the graph. *D*, echocardiographic images of control and RhoA cKO mice at the indicated time points. *E*–*H*, echocardiographic analyses of left ventricular ejection fraction (LVEF; *E*), LV end-diastolic diameter (LVDd; *F*), LV mass (*G*), and LV posterior wall thickness at end-diastole (LVPWd; *H*) in mice at 9, 13, 21, 29, 37, 45, and 53 weeks after birth. *I* and *J*, heart rate (HR; *I*) and systolic blood pressure (BP; *J*) measured by plethysmographic tail-cuff method in 9-, 21-, and 45-week-old mice. The data in each graph are shown as the mean ± SD. In (*A*) and (*E*–*J*) two-way ANOVA and one-way ANOVA were applied to compare the data between groups and weeks, respectively, and in (*C*), the data were analyzed by Kaplan–Meier method. ∗*p* < 0.05, ∗∗*p* < 0.01, and ∗∗∗*p* < 0.001 *versus* control; ^†^*p* < 0.05, ^††^*p* < 0.01, and ^†††^*p* < 0.001 *versus* week 9. cKO, conditional knockout.

RhoA cKO mice were healthy at birth with normal growth. The mice were similar in weight, and no obvious phenotypic abnormalities were observed at around 10 weeks after birth, compared with the littermate control mice (Fig. 1*A*). However, after 10 weeks of age, the body weight of RhoA cKO mice did not increase further and was significantly lower than that of control mice. RhoA expression was confirmed to be absent in cardiomyocytes from RhoA cKO mice (Fig. 1*B*). Strikingly, RhoA cKO mice experienced early death from around 30 weeks of age compared with control mice (Fig. 1*C*). To investigate the cause of early death in RhoA cKO mice, we assessed the cardiac function of these mice by echocardiography. The left ventricular ejection fraction of RhoA cKO mice was initially normal after birth, but it decreased significantly with age (Fig. 1, *D* and *E*). In addition to the lower left ventricular ejection fraction (LVEF), LV dilatation and increased LV mass without LV wall thickening during the experimental period were observed in the RhoA cKO hearts compared with the control hearts (Fig. 1, *F*–*H*), suggesting age-dependent cardiomyopathy caused by loss of RhoA in the heart. We also measured heart rate (HR) and blood pressure (BP). HR was similar between RhoA cKO mice and control mice, while RhoA cKO mice exhibited an age-dependent lower systolic BP than control mice (Fig. 1, I and *J*). Collectively, these results indicate the severe low cardiac output condition and an accelerated transition to heart failure in RhoA cKO mice, resulting in a shorter lifespan.

### Accelerated aging and fibrosis in the RhoA cKO heart

**Figure 2:**
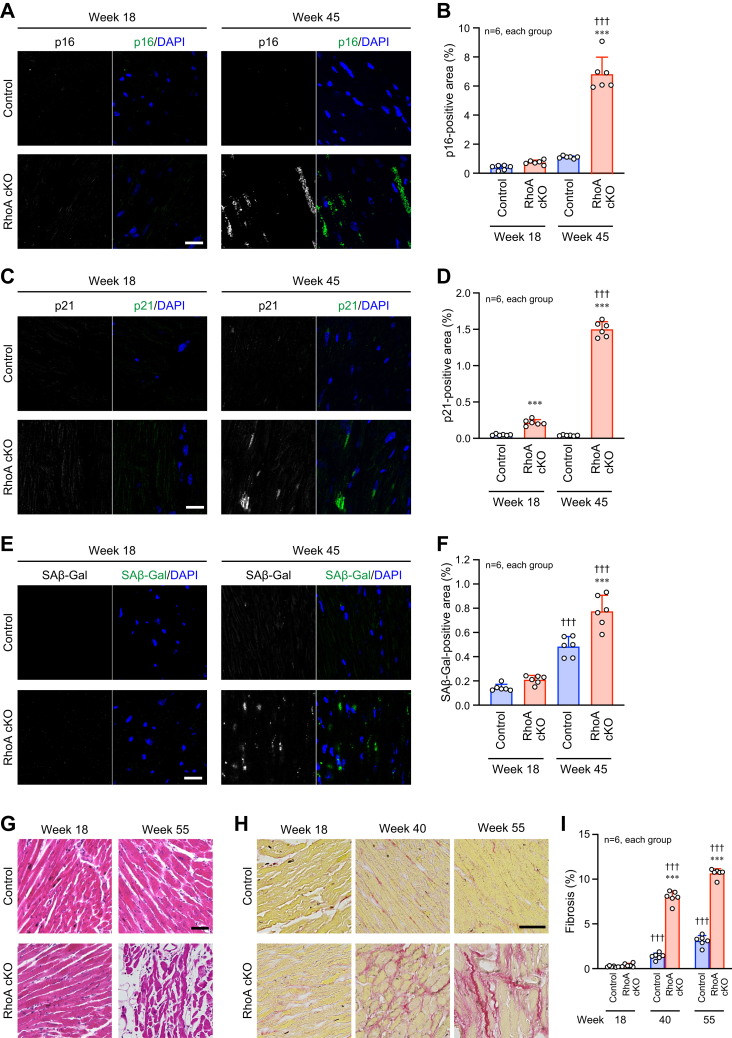
**Accelerated cardiac aging in RhoA cKO mice.***A*, *C*, and *E*, immunostaining for the senescence markers p16 (*A*), p21 (*C*), and senescence-associated β-galactosidase (SAβ-Gal; *E*) in the heart. Nuclei were counterstained with DAPI. Scale bars: 20 μm. *B*, *D*, and *F*, summary graphs of the percentage of positive area for each marker analyzed in (*A*, *C*, and *E*), respectively. *G*, H-E staining of the heart at the indicated time points. Scale bar: 50 μm. *H*, Picro-sirius red staining of the heart for the detection of fibrosis at the indicated time points. Scale bar: 50 μm. *I*, summary graph of the percentage of cardiac fibrosis. The data in each graph are shown as the mean ± SD. One-way ANOVA was applied to compare the data between groups in *B*, *D*, *F*, and *I*. ∗∗∗*p* < 0.001 *versus* control; ^†††^*p* < 0.001 *versus* week 18. cKO, conditional knockout; H-E, hematoxylin and eosin.

To explore how cardiac function rapidly declined with aging in RhoA cKO mice, we examined the progression of cardiac senescence using several markers because cardiac senescence impairs cardiac function (16, 17). Cellular senescence markers, including p16, p21, and senescence-associated β-galactosidase, were more highly detected in the RhoA cKO hearts than the control hearts (Fig. 2, *A*–*F*). Consistent with these results, the histological analysis by hematoxylin and eosin (H-E) staining revealed severe myocardial pathology, including increased myofiber disarray and interstitial space in LV of aged RhoA cKO mice (Fig. 2*G*). The RhoA cKO hearts also exhibited significantly augmented LV fibrosis (Fig. 2, H and I). In agreement with echocardiography, these results suggest that cardiac RhoA depletion accelerates cardiac aging and induces cardiac structural changes with abnormally increased fibrosis.

### Mitochondrial dysfunction and mitophagy dysregulation in the RhoA cKO heart

**Figure 3:**
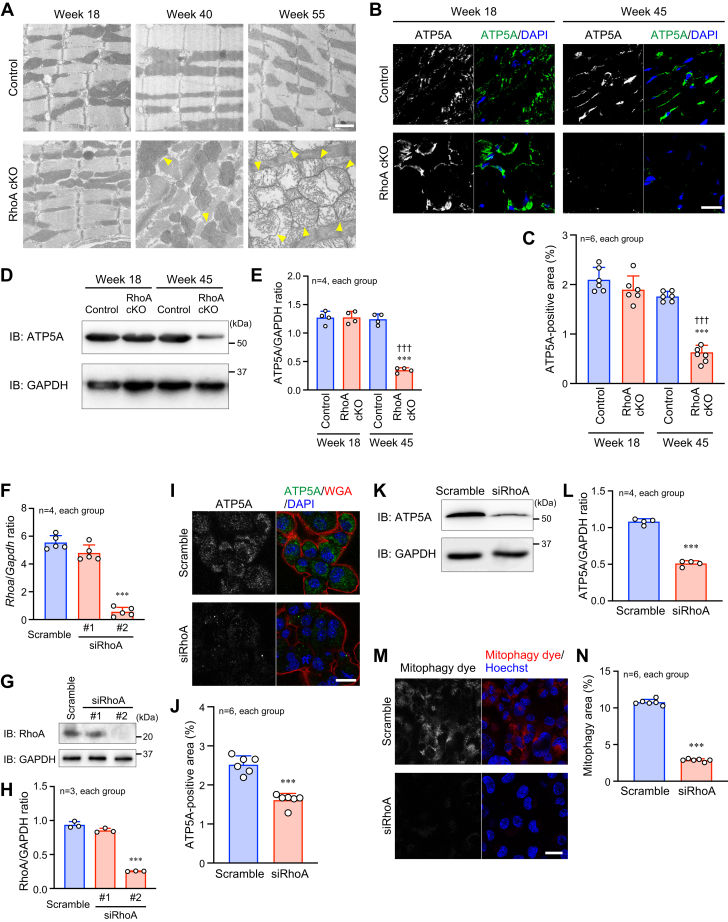
**Accumulation of dysfunctional mitochondria and mitophagy dysregulation in the RhoA cKO hearts.***A*, TEM images of the heart at the indicated time points. *Arrowheads* indicate swollen and severely damaged mitochondria. Scale bar: 1 μm. *B* and *D*, immunostaining (*B*) and Western blotting (*D*) for ATP5A in the mouse heart samples at the indicated time points. Nuclei were counterstained with DAPI (*B*), and GAPDH was blotted as the loading control (*D*). Scale bar in (*B*): 20 μm. *C* and *E*, summary graphs of the percentage of ATP5A-positive area (*C*) and the ATP5A/GAPDH band ratio (*E*) examined in (*B* and *D*), respectively. *F*, qPCR analysis of *Rhoa* gene expression in HL-1 cells after transfection of scramble RNA (Scramble), siRhoA #1, or siRhoA #2. *Gapdh* gene expression was used as the control. *G*, Western blotting of RhoA in HL-1 cells. *H*, summary graph of the RhoA/GAPDH band ratio in (*G*). *I* and *K*, immunostaining (*I*) and Western blotting (*K*) for ATP5A in HL-1 cells. The plasma membrane was stained with wheat germ agglutinin (WGA) in (*I*). Scale bar in (*I*): 20 μm. *J* and *L*, summary graphs of the percentage of ATP5A-positive area (*J*) and the ATP5A/GAPDH band ratio (*L*) examined in (*I* and *K*), respectively. *M*, fluorescence images of mitophagy in viable HL-1 cells after CCCP induction. Nuclei were counterstained with Hoechst. Scale bar: 20 μm. *N*, summary graph of the percentage of mitophagy area. The data in each graph are shown as the mean ± SD. Comparisons of the data between groups were performed using one-way ANOVA (*C* and *E*) or *t* test (*F*, *H*, *J*, *L*, and *N*). ∗∗∗*p* < 0.001 *versus* control or scramble; ^†††^*p* < 0.001 *versus* week 18. cKO, conditional knockout; TEM, transmission electron microscopy.

Mitochondrial dynamics in the heart are closely related to aging (7), and abnormal mitochondrial dynamics result in an insufficient energy supply in the heart, suppressing cardiac function (8, 10). Thus, we examined the morphology of the mitochondria in the heart by transmission electron microscopy (TEM). The mitochondria in the RhoA cKO hearts were severely damaged by aging, which occurred in parallel with the accumulation of many swollen and fragmented mitochondria with cristae disruption (Fig. 3*A*). We also found that the expression of ATP5A, a subunit of the mitochondrial ATP synthase, decreased in the heart of RhoA cKO mice compared with control mice (Fig. 3, *B*–*E*), validating the functionally defective mitochondria that resulted from RhoA knockout. Next, to examine the effect of RhoA on mitochondrial function in *in vitro* experiments, RhoA expression was knocked down in HL-1 cardiomyocytes. When two siRNAs for RhoA were transfected into HL-1 cells to check their efficiency for RhoA inhibition, siRhoA #2 significantly reduced RhoA expression, while siRhoA #1 did not. Thus, siRhoA #2 was used for further experiments (Fig. 3, *F*–*H*). Similar to the RhoA cKO hearts, ATP5A expression was significantly reduced in RhoA-knockdown HL-1 cardiomyocytes (Fig. 3, *I*–*L*).

Mitochondrial function and homeostasis are mainly regulated by (1) mitophagy and (2) fission and fusion (18, 19). Mitophagy is an important regulatory mechanism for clearing damaged mitochondria by proteasomal degradation. We first detected impaired mitophagy in siRhoA-treated HL-1 cells compared with scramble RNA-treated cells after exposure to carbonyl cyanide m-chlorophenyl hydrazone (Fig. 3, *M* and *N*). These results suggest defective mitophagy regulation in the absence of RhoA, leading to impaired removal and abnormal accumulation of damaged mitochondria in the heart in response to cardiac stress, such as aging. In contrast, the expression and phosphorylation of the mitochondrial fission marker Drp1 were not different between the RhoA cKO and control hearts or between RhoA-knockdown and control HL-1 cells (Fig. S1), suggesting that mitochondrial biogenesis in cardiomyocytes is normal, regardless of the absence of RhoA.

### Reduced Parkin expression and ubiquitinated mitochondrial proteins by loss of RhoA in cardiomyocytes

**Figure 4:**
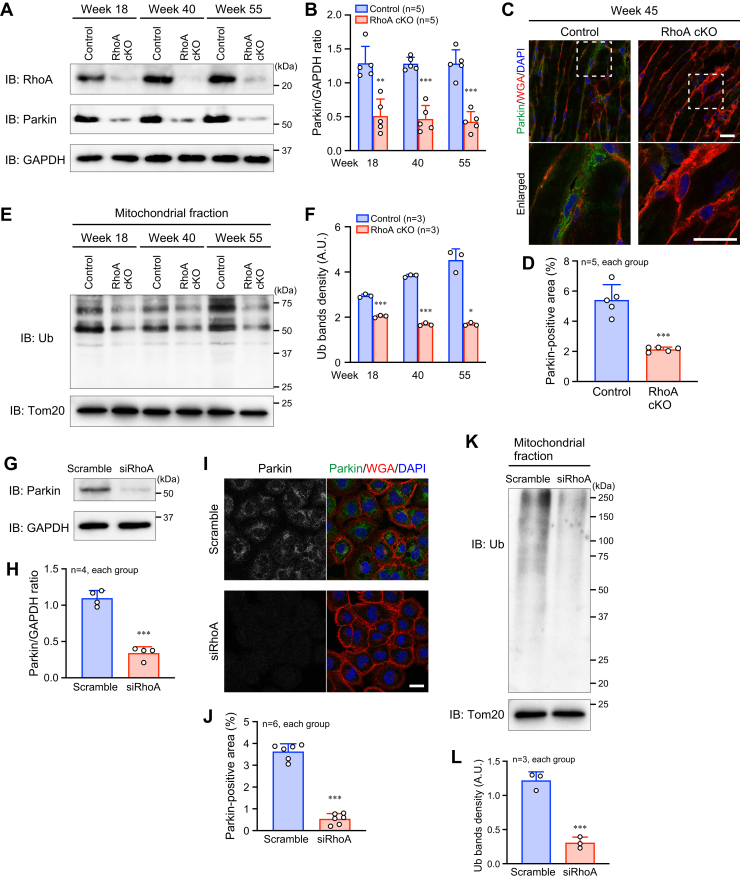
**Reduced Parkin expression in the RhoA cKO hearts.***A*, Western blotting for RhoA and Parkin in the heart at the indicated time points. GAPDH was blotted as the loading control. *B*, summary graph of the Parkin/GAPDH band ratio. *C*, immunostaining for Parkin in the hearts of 45-week-old mice. The plasma membrane and nuclei were counterstained with WGA and DAPI, respectively. Scale bar: 20 μm. *D*, summary graph of the percentage of Parkin-positive area. *E*, Western blotting for ubiquitin (Ub) in the mitochondrial fraction of the heart samples at the indicated time points. Tom20 was blotted as the loading control. *F*, summary graph of Ub bands density. A.U.: arbitrary unit. *G* and *I*, Western blotting (*G*) and immunostaining for Parkin (*I*) in HL-1 cardiomyocytes transfected with siRhoA and scramble RNA. *H* and *J*, summary graphs of the Parkin/GAPDH band ratio (*H*), and the percentage of Parkin-positive area (*J*) examined in (*G* and *I*), respectively. *K*, Western blotting for Ub in the mitochondrial fraction of HL-1 cells. Tom20 was blotted as the loading control. *L*, summary graph of Ub bands density. A.U.: arbitrary unit. The data in each graph are shown as the mean ± SD. Comparisons of the data between groups were performed using one-way ANOVA (*B* and *F*) or *t* test (*D*, *H*, *K*, and *L*). ∗*p* < 0.05, ∗∗*p* < 0.01, and ∗∗∗*p* < 0.001 *versus* control or scramble. cKO, conditional knockout; WGA, wheat germ agglutinin.

To delineate the mitochondrial abnormality and mitophagy dysregulation in cardiomyocytes with loss of RhoA, we focused on Parkin, an E3 ubiquitin (Ub) ligase, which mediates the ligation of Ub to the damaged mitochondria for proteasomal degradation (20). The loss of RhoA resulted in the reduction of Parkin expression with a decrease in ubiquitinated mitochondrial proteins in the hearts of younger (18-week-old) and older (55-week-old) mice (Fig. 4, *A*–*F*). Similarly, Parkin expression and ubiquitinated mitochondrial proteins in HL-1 cells were suppressed by RhoA knockdown (Fig. 4, *G*–*L*). These data suggest that RhoA plays a role in the expression of Parkin in cardiomyocytes, which regulates ubiquitination of mitochondrial proteins.

Parkin is phosphorylated, and its function is regulated by PTEN-induced putative kinase 1 (PINK1) (21). We then examined PINK1 expression in the presence and absence of RhoA in the mouse heart. PINK1 protein expression was almost identical between control and RhoA cKO hearts and was not different between young (18-week-old) and old (53-week-old) mice (Fig. S2, *A* and *B*). Similar to this, the gene expression of *Park6*, encoding PINK1, as well as the protein expression of PINK1 was not changed by RhoA knockdown in HL-1 cardiomyocytes, as shown by quantitative PCR and Western blotting (Fig. S2, *C*–*E*). Immunostaining of HL-1 cells also showed that the PINK1-positive area after siRhoA transfection was equal to that after scramble RNA transfection (Fig. S2, *F* and *G*). Thus, RhoA does not seem to affect PINK1 expression in the heart.

### RhoA-mediated N-Myc–Parkin pathway regulation

**Figure 5:**
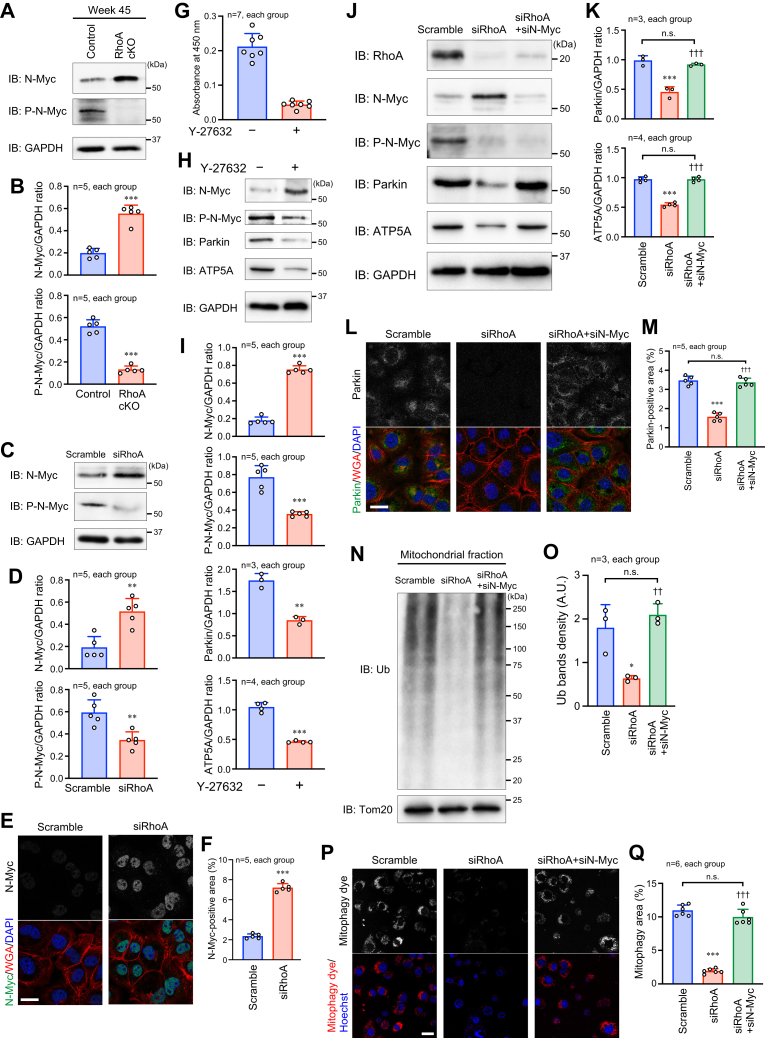
**RhoA-mediated Parkin expression in cardiomyocytes.***A* and *C*, Western blotting for N-Myc and phosphorylated N-Myc (P-N-Myc) in the hearts of 45-week-old mice (*A*) and HL-1 cardiomyocytes (*C*). GAPDH was blotted as the loading control. *B* and *D*, summary graphs of the N-Myc/GAPDH and P-N-Myc/GAPDH band ratios examined in (*A* and *C*), respectively. *E*, immunostaining for N-Myc in HL-1 cells. The plasma membrane and nuclei were counterstained with WGA and DAPI, respectively. Scale bar: 20 μm. *F*, summary graph of the percentage of N-Myc-positive area. *G*, ROCK kinase assay in HL-1 cells with or without a ROCK inhibitor Y-27632 for 1 h. *H*, Western blotting for the indicated molecules in HL-1 cells treated with or without Y-27632 for 1 h. *I*, summary graphs of the ratio for the band density of each molecule to that of GAPDH, which was examined in (*H*). *J*, Western blotting for the indicated molecules in HL-1 cells transfected with siRhoA, siRhoA+siN-Myc, or scramble RNA as the control. *K*, summary graphs of the Parkin/GAPDH and ATP5A/GAPDH band ratios. *L*, immunostaining for Parkin in HL-1 cells. Scale bar: 20 μm. *M*, summary graph of the percentage of Parkin-positive area in (*L*). *N*, Western blotting for Ub in the mitochondrial fraction of HL-1 cells. Tom20 was blotted as the loading control. *O*, summary graph of Ub bands density. A.U.: arbitrary unit. *P*, fluorescence images of mitophagy in viable cultured HL-1 cells after CCCP induction. Nuclei were counterstained with Hoechst. Scale bar: 20 μm. *Q*, summary graph of the percentage of mitophagy area. The data in each graph are shown as the mean ± SD. In (*C*, *E*, *J*, *L*, *N*, and *P*), HL-1 cells were used for experiments at 48 h after siRNA transfection. Comparisons of the data between groups were performed using *t* test (*B*, *D*, *F*, and *H*) or one-way ANOVA (*J*, *L*, *O*, and *Q*). ∗*p* < 0.05, ∗∗*p* < 0.01, and ∗∗∗*p* < 0.001 *versus* control or scramble; ^††^*p* < 0.01 and ^†††^*p* < 0.001 *versus* siRhoA. CCCP, carbonyl cyanide m-chlorophenyl hydrazone; ROCK, Rho kinase; Ub, ubiquitin; WGA, wheat germ agglutinin.

N-Myc is a negative transcription factor for the *Parkin* gene expression (22), and the expression of N-Myc is reduced by phosphorylation-dependent degradation (23). We determined the endogenous expression of N-Myc in both the mouse heart and HL-1 cells. By depletion of RhoA, the expression of N-Myc was increased (Fig. 5, *A*–*F*), together with a remarkable reduction of its phosphorylation (Fig. 5, *A*–*D*), indicating an inverse correlation between N-Myc and Parkin expressions. To further examine how RhoA regulates N-Myc phosphorylation, we focused on Rho kinase (ROCK), which is an effector of RhoA (14). ROCK in HL-1 cells was confirmed to be inhibited by treatment with a ROCK inhibitor Y-27632 (Fig. 5*G*). In the presence of Y-27632, N-Myc phosphorylation was decreased, and N-Myc expression was increased, resulting in the reduction of Parkin and ATP5A expressions (Fig. 5, *H* and *I*).

We next examined whether N-Myc deletion in RhoA-deficient cardiomyocytes could rescue Parkin expression. When N-Myc was knocked down by transfection of siN-Myc in RhoA-knockdown HL-1 cells, the reduced expression of Parkin in RhoA-knockdown cells was restored (Fig. 5, *J*–*M*). Similarly, the attenuated ATP5A expression in RhoA-knockdown HL-1 cells was recovered by the additional N-Myc knockdown (Fig. 5, *J* and *K*). The ubiquitination of mitochondrial proteins was also recovered by siN-Myc transfection in RhoA-knockdown HL-1 cells (Fig. 5, *N* and *O*). Furthermore, immunofluorescent microscopy confirmed the recovery of mitophagy by the additional N-Myc knockdown (Fig. 5, *P* and *Q*). Taken together, our findings suggest that N-Myc functions downstream of RhoA as a negative regulator of Parkin expression and that N-Myc expression is inhibited by the RhoA–ROCK-mediated N-Myc phosphorylation, leading to the sufficient Parkin expression for maintenance of cardiomyocyte mitophagy.

### Restoration of mitophagy and cardiac function by supplementation of Parkin expression in the RhoA-depleted cardiomyocytes

**Figure 6:**
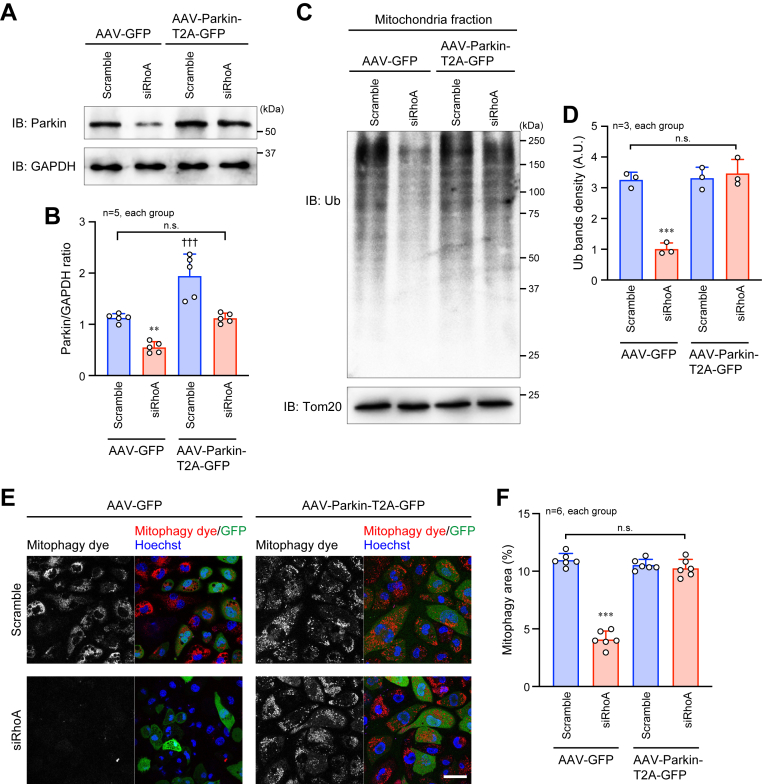
**Restoration of mitochondrial function in RhoA-knockdown HL-1 cardiomyocytes by AAV-Parkin infection.***A*, Western blotting for Parkin in HL-1 cardiomyocytes infected with AAV-Parkin-T2A-GFP or AAV-GFP as the control. GAPDH was blotted as the loading control. *B*, summary graph of the Parkin/GAPDH band ratio. *C*, Western blotting for Ub in the mitochondrial fraction of HL-1 cells. Tom20 was blotted as the loading control. *D*, summary graph of Ub bands density. A.U.: arbitrary unit. *E*, fluorescence images of mitophagy in viable cultured HL-1 cells after CCCP induction. Nuclei were counterstained with Hoechst. Scale bar: 20 μm. *F*, summary graph of the percentage of mitophagy area. The data in each graph are shown as the mean ± SD. One-way ANOVA was used to compare the data between groups. ∗∗*p* < 0.01 and ∗∗∗*p* < 0.001 *versus* Scramble; ^†††^*p* < 0.001 *versus* AAV-GFP. AAV, adeno-associated virus; CCCP, carbonyl cyanide m-chlorophenyl hydrazone; GFP, green fluorescent protein.

To demonstrate the essential effects of Parkin on the rescue of mitophagy and cardiac function in the RhoA cKO hearts and RhoA-knockdown cardiomyocytes, we used the adeno-associated virus (AAV) serotype 6 gene transfer system to introduce the *Parkin* gene in cardiomyocytes. First, we infected AAV-Parkin-T2A-green fluorescent protein (GFP) and control AAV-GFP into HL-1 cells and examined how the infection increased Parkin expression. AAV-Parkin-T2A-GFP infection recovered the siRhoA-mediated decrease in Parkin expression back to the basal level (Fig. 6, *A* and *B*). Similarly, AAV-Parkin-T2A-GFP restored mitochondrial protein ubiquitination and mitophagy (Fig. 6, *C*–*F*). In the fluorescence microscopy, we found that all of the HL-1 cells were infected with AAV-Parkin-T2A-GFP or AAV-GFP as monitored by GFP fluorescence, although the level of GFP fluorescence was variable in each cell (Fig. 6*E*).

**Figure 7:**
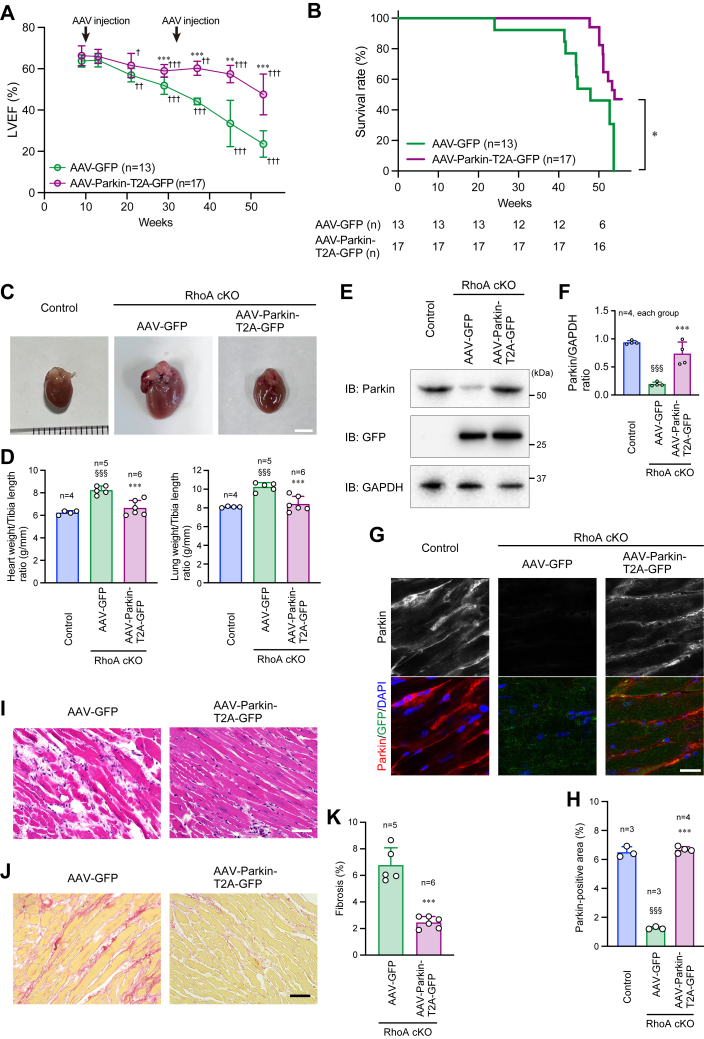
**Improved cardiac function and increased lifespan in RhoA cKO mice after intravenous administration of AAV-Parkin.***A*, LVEF analyzed by echocardiography in 9-, 13-, 21-, 29-, 37-, 45-, and 53-week-old mice. AAV-Parkin-T2A-GFP or AAV-GFP as the control was injected into RhoA cKO mice through the tail vein twice (10 and 32 weeks after birth). *B*, Kaplan–Meier survival curve of RhoA cKO mice injected twice with AAV-Parkin-T2A-GFP or AAV-GFP. The number of mice (n) in each group at every 10 weeks is indicated below the graph. *C*, external appearance of the hearts extracted from 53-week-old mice. Scale bar: 5 mm. *D*, heart and lung weight in 53-week-old mice, which was normalized to tibia length. *E*, Western blotting for Parkin and GFP in the hearts of 53-week-old mice. GAPDH was blotted as the loading control. *F*, summary graph of the Parkin/GAPDH band ratio. *G*, immunostaining for Parkin in the hearts of 53-week-old mice. Nuclei were counterstained with DAPI. Scale bar: 20 μm. *H*, summary graph of the percentage of Parkin-positive area. *I* and *J*, H-E staining and Picro-sirius red staining of the hearts from 53-week-old mice. Scale bars: 50 μm. *K*, summary graph of the percentage of cardiac fibrosis examined in (*J*). The data in each graph are shown as the mean ± SD. In (*A*), two-way ANOVA and one-way ANOVA were applied to compare the data between groups and weeks, respectively, and in (*C*), the data were analyzed using the Kaplan–Meier method. One-way ANOVA (*D*, *F*, and *H*) or *t* test (*K*) was used to compare the data between groups. ∗*p* < 0.05, ∗∗*p* < 0.01, and ∗∗∗*p* < 0.001 *versus* AAV-GFP; ^†^*p* < 0.05, ^††^*p* < 0.01, and ^†††^*p* < 0.001 *versus* Week 9; ^§§§^*p* < 0.001 *versus* control. AAV, adeno-associated virus; cKO, Conditional knockout; GFP, green fluorescent protein; H-E, hematoxylin and eosin; LVEF, Left ventricular ejection fraction.

Next, we examined the *in vivo* function of AAV-Parkin-T2A-GFP in RhoA cKO mice by intravenous injection of AAV through the tail vein. When AAV-Parkin-T2A-GFP was administered in control mice to assess the *in vivo* efficiency of the AAV-mediated *Parkin* gene transfer, Parkin expression was increased in the heart compared with the administration of AAV-GFP (Fig. S3, *A* and *B*). In contrast, the increase was not observed in the brain (Fig. S3*A*), confirming adequate gene transfer by the AAV serotype 6 system. Four weeks after AAV infection in mice, cardiac function was unchanged, as evaluated by LVEF and hemodynamics, such as HR and systolic BP (Fig. S3*C*). This suggests that *Parkin* gene was safely transferred by AAV *in vivo*. Because Parkin expression in the heart was restored for approximately 25 weeks after AAV-Parkin-T2A-GFP administration in RhoA cKO mice (Fig. S3*D*), we injected AAV in RhoA cKO mice twice (10 and 32 weeks after birth) for a total 1-year (53 weeks) observation period. After the injection of AAV-Parkin-T2A-GFP, the deterioration of LVEF was attenuated, and the lifespan was prolonged compared with mice injected with AAV-GFP (Fig. 7, *A* and *B*). Mice were sacrificed at around 55 weeks after birth. The heart was enlarged in RhoA cKO mice injected with control AAV-GFP due to heart failure, which was clearly recovered by AAV-Parkin-T2A-GFP injection (Fig. 7, *C* and *D*). Similarly, the lung weight, which was also increased by heart failure-induced pulmonary edema in RhoA cKO mice, was reduced by AAV-Parkin-T2A-GFP injection (Fig. 7*D*). The treatment maintained the expression of Parkin in the RhoA cKO hearts, and the results were comparable to those of the control hearts (Fig. 7, *E*–*H*). H-E staining showed an improvement of the severe myocardial damage in the RhoA cKO hearts after AAV-Parkin-T2A-GFP injection (Fig. 7*I*). The increased cardiac fibrosis in the RhoA cKO hearts was also attenuated by the injection (Fig. 7, *J* and *K*).

**Figure 8:**
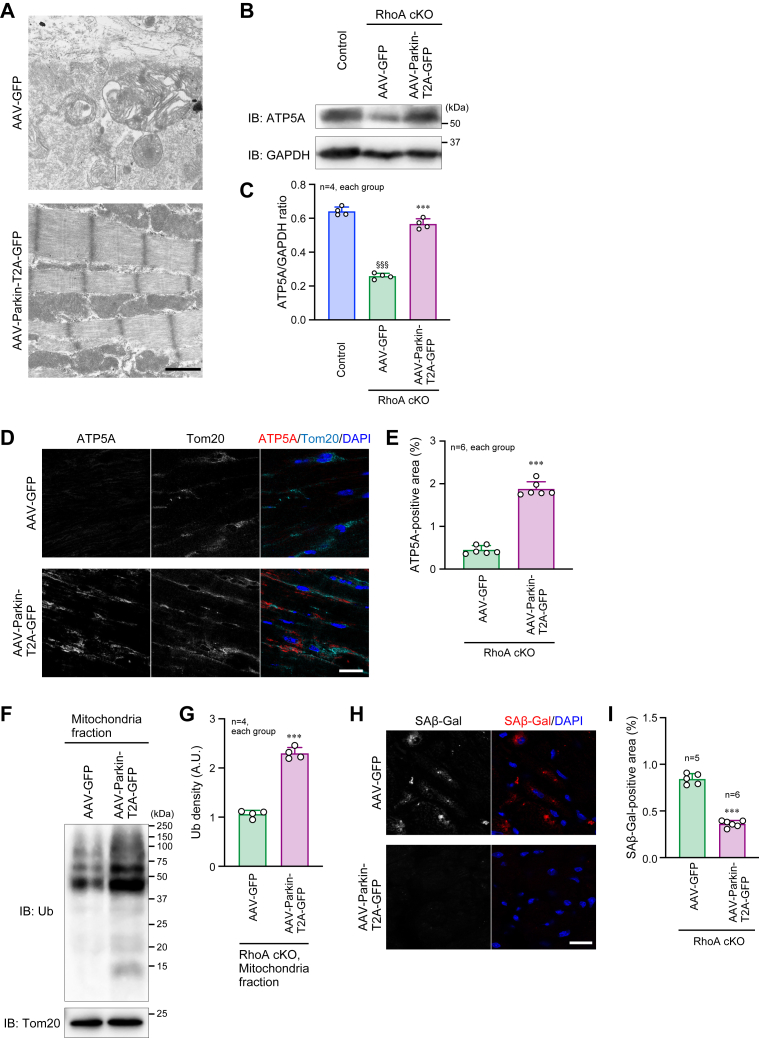
**Clearance of damaged mitochondria and improvement of earlier senescence in RhoA cKO mice after AAV-Parkin injection.***A*, TEM images of the hearts from 53-week-old mice after intravenous injection of AAV-Parkin-T2A-GFP or AAV-GFP twice (10 and 32 weeks after birth). Scale bar: 1 μm. *B*, Western blotting for ATP5A in the hearts from 53-week-old mice. GAPDH was blotted as the control. *C*, summary graph of the ATP5A/GAPDH band ratio examined in (*B*). *D*, co-immunostaining for ATP5A and Tom20 in the hearts from 53-week-old mice. Nuclei were counterstained with DAPI. Scale bar: 20 μm. *E*, summary graph of the percentage of ATP5A-positive area examined in (*D*). *F*, Western blotting for Ub in the mitochondrial fraction of the hearts from 53-week-old mice. Tom20 was blotted as the loading control. *G*, summary graph of Ub bands density. A.U.: arbitrary unit. *H*, Immunostaining for a senescence marker SAβ-Gal in the hearts from 53-week-old mice. Scale bar: 20 μm. *I*, summary graph of the percentage of SAβ-Gal-positive area. The data in each graph are shown as the mean ± SD. One-way ANOVA (*C*) or *t* test (*E*, *G*, and *I*) was used to compare the data between groups. ∗∗∗*p* < 0.001 *versus* AAV-GFP; ^§§§^*p* < 0.001 *versus* control. AAV, adeno-associated virus; cKO, conditional knockout; GFP, green fluorescent protein; Ub, ubiquitin.

Further ultrastructural analysis using TEM revealed a remarkable reduction of damaged mitochondria in the RhoA cKO hearts after treatment with AAV-Parkin-T2A-GFP (Fig. 8*A*). This was justified by the restored expression of ATP5A and the increase in mitochondrial protein ubiquitination in the AAV-Parkin-T2A-GFP-treated RhoA cKO hearts (Fig. 8, *B*–*G*). Cellular senescence in RhoA cKO cardiomyocytes was also suppressed by AAV-Parkin-T2A-GFP treatment (Fig. 8, *H* and *I*). These findings suggest that Parkin, as the downstream molecule of RhoA, could compensate for RhoA deficiency by maintaining mitochondrial homeostasis, resulting in the prevention of age-related acceleration of cardiac dysfunction and heart failure in the absence of RhoA.

Considering the clinical implication of Parkin expression supplementation in patients with heart failure caused by reduction or loss of RhoA, we additionally examined the effect of the *Parkin* gene transfer on RhoA cKO mice when cardiac function was mildly impaired. After AAV-Parkin-T2A-GFP was administered once in 30-week-old RhoA cKO mice, the impairment of LVEF tended to be prevented for 15 weeks after the administration (Fig. S4*A*), and the lifespan was significantly prolonged compared with after AAV-GFP administration (Fig. S4*B*). Parkin expression in 50-week-old mice after a single AAV-Parkin-T2A-GFP administration was higher than after AAV-GFP administration, whereas the expression was lower than after AAV-Parkin-T2A-GFP administration twice (Fig. S4, *C*–*F*). The histological analysis showed that dysregulation of myocardial tissue and the degree of fibrosis were similar between mice treated with AAV-Parkin-T2A-GFP and mice treated with AAV-GFP (Fig. S4, *G*–*I*). However, cardiac senescence determined by senescence-associated β-galactosidase and mitochondrial function evaluated by ATP5A expression were significantly improved after a single administration of AAV-Parkin-T2A-GFP compared with AAV-GFP administration (Fig. S4, *J*–*O*), suggesting the benefit of the *Parkin* gene transfer in RhoA cKO mice even after mild heart failure begins.

### Reduced RhoA expression in aged patients with idiopathic dilated cardiomyopathy

**Figure 9:**
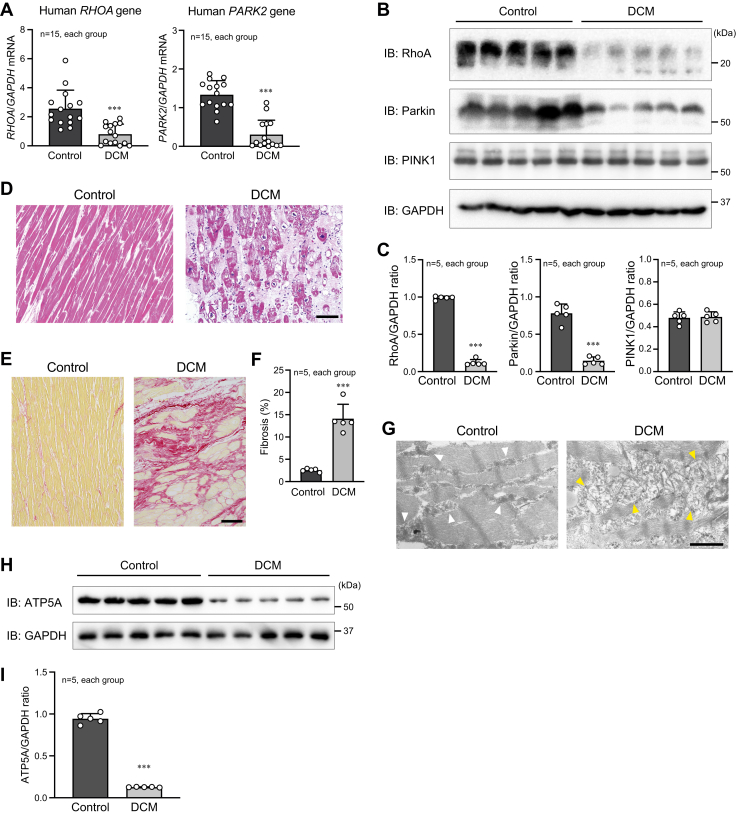
**Reduced RhoA and Parkin expressions and impaired mitochondrial function in patients with idiopathic DCM.***A*, qPCR analysis of gene expressions of *RHOA* and *PARK2*, which encodes Parkin, in the human heart. Control heart samples were obtained from subjects who died accidentally, and DCM heart samples were obtained at the time of heart transplantation. *GAPDH* gene expression was used as the control. *B*, Western blotting for RhoA, Parkin, and PINK1 in the human heart. GAPDH was blotted as the loading control. *C*, summary graphs of the band ratios of RhoA, Parkin, and PINK1 to GAPDH examined in (*B*). *D* and *E*, H-E staining and Picro-sirius red staining of the human heart. Scale bar: 100 μm. *F*, summary graph of the percentage of cardiac fibrosis examined in (*E*). *G*, TEM images of the human heart. *White* and *yellow arrowheads* indicate normal and disrupted mitochondria, respectively. Scale bar: 1 μm. *H*, Western blotting for ATP5A in the human heart. *I*, summary graphs of the ATP5A/GAPDH ratio examined in (*H*). The data in each graph are shown as the mean ± SD. Comparisons of the data between the groups were conducted using *t* test. ∗∗∗*p* < 0.001 *versus* control. DCM, dilated cardiomyopathy; H-E, hematoxylin and eosin; PINK1, PTEN-induced putative kinase 1.

There is no definitive knowledge about the RhoA expression in aged patients who suffer from heart failure without known hereditary gene mutations. In this context, we examined the cardiac RhoA expression in the heart samples obtained from adult patients with severe heart failure caused by idiopathic dilated cardiomyopathy (DCM). The heart samples were obtained at the time of heart transplantation. The clinical characteristics of the patients are shown in Table 1. Because all patients underwent LV assist device (LVAD) implantation prior to heart transplantation, the data in Table 1 were obtained just before LVAD implantation. The average period between LVAD implantation and heart transplantation was 4.4 ± 0.9 years. Cardiac RhoA expression was significantly decreased in patients with idiopathic DCM compared with control subjects (average age: 38.5 ± 10.8 years; male/female (n): 12/3) who died accidentally without cardiovascular diseases (Fig. 9, *A*–*C*). Concomitant with these results, the significant reduction of Parkin expression was observed in the hearts of patients with DCM (Fig. 9, *A*–*C*). These findings may validate our hypothesis that reduced RhoA expression in the heart attenuates Parkin expression, not only in mice but also in humans. In contrast to RhoA and Parkin expressions, PINK1 expression in the heart was almost equal between DCM patients and control subjects (Fig. 9, *B* and *C*). We confirmed the severe myocardial damage and fibrosis in patients with DCM by histological analysis (Fig. 9, *D*–*F*). TEM also detected an abundance of disrupted mitochondria in the hearts of patients with DCM compared with control subjects (Fig. 9*G*). Finally, we found the significant decrease in ATP5A expression in the hearts of patients with DCM (Fig. 9, H and I). These results support our conclusion that RhoA plays a role in cardiac mitochondrial function *via* Parkin and that the defect of RhoA expression results in mitophagy dysregulation, leading to accelerated cardiac senescence and heart failure.

**Table 1:** Clinical characteristics of DCM patients

Total (n)	15
Age (year)	42.2 ± 11.6
Male/Female (n)	11/4
BMI (kg/m^2^)	21.9 ± 3.3
Hemoglobin (g/dl)	12.5 ± 1.6
Blood serum sample data	
Albumin (g/dl)	3.7 ± 0.7
LDH (IU/l)	227 ± 49
T-Bil (mg/dl)	1.4 ± 0.7
Creatinine (mg/dl)	1.01 ± 0.19
BNP (pg/ml)	881 ± 622
Echocardiography	
LVDd (mm)	74 ± 10
LAD (mm)	54 ± 10
LVEF (%)	16 ± 8

The data are shown as the mean ± SD.

Abbreviations: BMI, body mass index; BNP, brain natriuretic peptide; LDH, lactate dehydrogenase; T-Bil, total bilirubin.

## Discussion

This study provides an important insight into the function of RhoA in the aging heart, as well as the molecular mechanism by which RhoA regulates cardiac function through Parkin-mediated mitochondrial homeostasis. RhoA cKO mice showed earlier death from around 30 weeks of age and a dramatic reduction of LVEF with accelerated senescence and age-dependent cardiac fibrosis. Concomitant with the severe deterioration of cardiac function, we found that loss of RhoA in the heart induced excess accumulation of severely damaged mitochondria in cardiomyocytes. In patients with idiopathic DCM who had no hereditary gene mutations, both RhoA and Parkin expressions in the heart were markedly reduced, and the morphology of the cardiac mitochondria was disturbed. This suggests that RhoA has cardioprotective effects and is crucial for the maintenance of healthy mitochondria, resulting in the prevention of heart failure with aging.

In support of our findings, another research group has recently reported that in myocardial infarction, cardiac RhoA signaling plays a role in mitochondrial quality control by regulating the function and expression of Parkin and PINK1, a protein kinase that phosphorylates and activates Parkin (24). In our study, we further revealed the mechanism of RhoA in the regulation of Parkin expression through N-Myc in cardiomyocytes. N-Myc is a member of the Myc family. It is a transcription factor that is critically involved in diverse physiological and pathological events, including neuronal development and tumor progression (25, 26). This protein binds to the E-box motif at the *Parkin* transcription initiation site and transcriptionally inhibits Parkin expression in neuroblastoma cell lines (22). In this study, we observed downregulation and upregulation of N-Myc expression in RhoA-intact and RhoA-depleted cardiomyocytes, respectively. N-Myc expression has also been shown to be suppressed by its phosphorylation and subsequent ubiquitination (23, 27). GSK-3β was identified to be a kinase that phosphorylates N-Myc, but other kinases that contribute to N-Myc phosphorylation have not been well documented. Using a ROCK inhibitor Y-27632, we discovered that ROCK, which is an effector of RhoA, functions as a kinase that phosphorylates N-Myc to reduce its expression. Thus, we propose that the RhoA–ROCK axis negatively regulates N-Myc to maintain sufficient Parkin expression in cardiomyocytes.

As for ROCK, there are two isoforms ROCK1 and ROCK2, and the disruption of both ROCK isoforms has been reported to be cardioprotective by promoting autophagy and reducing cardiac fibrosis during aging (28). ROCKs are well-known effectors of RhoA, while other proteins, such as mDia, also function downstream of RhoA. Inhibition of mDia in the heart markedly suppressed the cardiac function and induced heart failure (29). In addition, a single deletion of ROCK2 in cardiomyocytes was profibrotic and reduced autophagy (28), suggesting that only ROCK1 deletion is favorable for cardiomyocytes and overwhelms the ROCK2 deletion-mediated deteriorative cardiac phenomena. Collectively, because RhoA regulates a variety of molecules including ROCKs, it might be reasonable that the phenomena observed in RhoA cKO mice are different from those in mice in which double cardiac ROCKs are ablated.

Mutations in the *Parkin* gene are intimately related to familial Parkinson’s disease (PD) (30). PD is the common neurodegenerative disorder that involves loss of dopaminergic neurons in the substantia nigra (31, 32). In addition to neuronal system dysfunction in PD, PD is associated with the risk of cardiovascular disease, including congestive heart failure (33). Although the role of Parkin in the brain has been extensively studied (34, 35), the understanding of Parkin regulation in the aging heart downstream of RhoA remains elusive. Mitophagy is the system that clears the damaged mitochondria in various cell types, including cardiomyocytes, and is fundamental for constitutive mitochondrial housekeeping to maintain cardiac homeostasis and prevent heart failure (36). Several reports have demonstrated the pathophysiological importance of mitophagy in the heart, in which Parkin exerts cardioprotection in response to ischemic stress (37, 38). In addition, PINK1 contributes to the maintenance of cardiac function because PINK1 knockout mice develop LV dysfunction and pathological cardiac hypertrophy with impaired mitochondrial function (39). Although the present study showed that loss of RhoA in cardiomyocytes attenuated Parkin expression, PINK1 expression was not changed. Moreover, the mitochondrial fission marker Drp1 and its phosphorylated form were not disturbed in the RhoA cKO hearts and RhoA-knockdown HL-1 cells. These findings suggest that RhoA specifically regulates Parkin in cardiomyocytes, independent of PINK1, and that it does not affect mitochondrial biogenesis.

Parkin is a cytosolic E3 Ub ligase that selectively ubiquitinates proteins located on dysfunctional mitochondria for mitophagy (40, 41). To prevent unnecessary cell death, dysfunctional mitochondria, which are harmful to cells, should be cleared, and mitophagy is one of the systems responsible for this clearance. In our study, the hearts from 18-week-old RhoA cKO mice had normal mitochondria, while the hearts from 55-week-old RhoA cKO mice had swollen and disorganized mitochondria with broken cristae, which was quite different from the hearts from 55-week-old control mice that had morphologically normal mitochondria. Our data suggest that RhoA deficiency in the heart causes a defect in the clearance of dysregulated mitochondria due to reduced Parkin expression. Similar to RhoA cKO mice in the present study, young 12-week-old Parkin^−/−^ mice had normal cardiac function under baseline conditions in a previous study. However, Parkin^−/−^ mice were quite sensitive to the cardiac stress induced by myocardial infarction (42). According to another previous study, mitochondrial DNA mutations in mice accelerated cardiac aging, and overexpression or deletion of Parkin in the mice did not rescue or worsen the cardiac phenotype (43). These results differ from ours; however, different mouse models may demonstrate different degrees of mitochondrial damage and different regulatory mechanisms to compensate for the defect in mitophagy in the aged heart, which may affect the rate of transition to cardiomyopathy.

One advantage of our study is that we demonstrated the significant reduction of both RhoA and Parkin expressions in patients with DCM compared with normal subjects. Although it might be difficult to strictly identify which of RhoA or Parkin reduction is the primary and specific cause of DCM, it is possible to interpret that RhoA is involved in cardiac homeostasis cooperatively with Parkin. Mitochondrial morphology and function as well as mitophagy were disturbed in patients with DCM in the present study. Furthermore, several novel functions of RhoA, which are mediated by Parkin, were observed not only in the mouse heart but also in the human heart. To date, several gene mutations associated with heart failure have been listed (44, 45). Loss or mutation of the *RHOA* and *PARK2* genes can be added to the list in line with the findings from our and other research groups.

In conclusion, we showed the functional role of RhoA in regulating Parkin expression through ROCK and N-Myc and Parkin-dependent mitophagy for the clearance of damaged mitochondria in the heart, resulting in the maintenance of mitochondrial homeostasis and prevention of cardiac senescence (Fig. S5). Thus, we conclude that loss of RhoA in the heart induces heart failure due to early cardiac senescence and cardiomyopathy. Further understanding of RhoA signaling in the aged heart will help to develop future therapies for the prevention and treatment of heart failure.

## Experimental procedures

### Generation of RhoA cKO mice

RhoA-floxed mice (RhoA^fl/fl^: C57BL/6 background), in which exon 3 of the *Rhoa* gene was flanked by loxP sites, were generated and used in our previous study (46). The mice were then mated with C57BL/6 mice expressing Cre recombinase under the control of the α-myosin heavy chain promoter (Myh6-Cre; Jackson Laboratory) to generate cardiomyocyte-specific RhoA cKO mice. In the Myh6-Cre mice, Cre exerts its recombination activity specifically in cardiomyocytes but not in other tissues such as the liver, lung, skeletal muscle, and spleen (47), and the recombinase functions from embryonic day 9.5 (48). Mice harboring RhoA^fl/fl^ alleles alone were used as controls. The mice were housed in specific pathogen-free conditions at the Research Centre for Animal Life Science of Shiga University of Medical Science. All animal protocols were in accordance with institutional guidelines, including Animal Research Reporting of *In Vivo* Experiments (ARRIVE) guidelines, and were approved by the Animal Care and Use Committee of Shiga University of Medical Science (No. 2020-9-8).

### Human heart sample collection

All protocols using human heart samples were approved by the Research Ethics Committee of Osaka University Graduate School of Medicine and Shiga University of Medical Science and conformed to the principles of the Declaration of Helsinki. Heart tissues were obtained from (1) subjects who died accidentally without cardiovascular diseases and were sent to Division of Legal Medicine, Shiga University of Medical Science, for forensic autopsy and (2) patients with idiopathic DCM at the time of heart transplantation during the period of November 2017 through June 2021. All of the patients provided written informed consent for the use of heart tissues in this study.

### Cell culture, siRNA transfection, and plasmid transfection

HL-1 cells (a gift from Dr Ayako Takeuchi, Faculty of Medical Science, University of Fukui, Japan) were cultured in Claycomb Medium supplemented with 10% fetal bovine serum, 2 mmol/l L-glutamine, 100 μg/ml penicillin/streptomycin, and 0.1 mmol/l norepinephrine (Nacalai Tesque) as previously described (49). siRhoA, siN-Myc, and negative control (scramble) RNA were synthesized using the CUGA7 *in vitro* Transcription Kit (Nippon Gene). The siRNA sequences were as follows: siRhoA #1 (5′-GACAUGCUUGCUCAUAGUCUU-3′), siRhoA #2 (5′-GCAGAGAUAUGGCAAACAG-3′), siN-Myc (5′-GCUCUUGCGGCCAGUAUUA-3′), and scramble RNA (5′-CAGUCGCGUUUGCGACUGG-3′). HL-1 cells were plated 24 h prior to siRNA transfection (2 μmol/l each), which was introduced using Lipofectamine RNAiMAX reagent (Invitrogen). After 48 h of siRNA transfection, HL-1 cells were used for experiments. The duration of the treatment with 10 μmol/l Y-27632 was 1 h. As for the transfection of plasmids into the cells, Lipofectamine 2000 reagent (Invitrogen) was used.

### Echocardiographic analysis

Echocardiography was performed on 9-, 13-, 21-, 29-, 37-, 45-, and 53-week-old mice. Mice were anesthetized with isoflurane/air mix (induced at 2% and maintained at ∼1% by isoflurane). LV dimension and cardiac function were assessed by transthoracic ultrasonography using the Vevo 2100 system (VisualSonics Inc). Mouse hearts were imaged in the two-dimensional parasternal long-axis (B-mode) and short-axis (M-mode) for cardiac systole evaluation. All measurements of cardiac anatomic and functional parameters were as described previously (50).

### BP measurement

Arterial BP and HR of conscious mice were assessed using the noninvasive plethysmographic tail-cuff method (model BP-98-AL; Softron). Mice were weighed and warmed at 37 °C in a cylindrical thermostat supplemented on the BP-98-AL machine before and during the assessment. Measurements were taken at 2-min intervals, and an average of five BP and HR measurements was taken as the true BP and HR of each mouse, respectively.

### Histological analysis of the heart

Fresh mouse and human hearts were fixed with 4% paraformaldehyde and 10% formaldehyde, respectively, followed by embedding in paraffin blocks overnight. Otherwise, the hearts were frozen in water-soluble medium (Surgipath FCS22; Leica Biosystems) in liquid nitrogen. Formalin-fixed paraffin–embedded heart tissues were sectioned at 4-μm thickness using a microtome (Leica Biosystems). The frozen heart tissues were sectioned at a thickness of 10 μm using a cryostat (Leica CM1800; Leica Biosystems) at −20 °C. The sections were layered on poly-L-lysine-coated slides. The formalin-fixed paraffin–embedded heart sections were deparaffinized before being subjected to H-E or Picro-Sirius red staining (51).

### Confocal microscopy

Cells on poly-L-lysine-coated cover slides were fixed with 4% paraformaldehyde and permeabilized with 0.2% Triton X-100 in phosphate-buffered saline (PBS) for 30 min at 37 °C. Primary antibodies were applied in 2% bovine serum albumin (BSA) plus 1% or 3% skimmed milk in PBS overnight, followed by a 1-h incubation with the fluorescent dye-labeled secondary antibody. The cells were imaged using the Leica SP8 X confocal microscope (Leica Microsystems). Similar staining techniques were performed on cross-sections of the frozen hearts. The percentage of positive area in the images was quantified using ImageJ software (National Institute of Health). After converting the composite fluorescent image (with three colors) into individual RGB images, the individual threshold level for each fluorescent marker was determined to generate the percentage of fluorescence positive area for each marker.

### TEM

Mouse and human hearts were fixed with 2.5% glutaraldehyde in 0.1 mol/l cacodylate buffer; postfixed in 1% osmium tetroxide; treated with 0.5% tannic acid, and 1% sodium sulfate; cleared in 2-hydroxypropyl methacrylate; and embedded in LX112 (Ladd Research). Sections were mounted on copper slot grids coated with parlodion and stained with uranyl acetate and lead citrate for examination on the H-7500 electron microscope (Hitachi High-Tech Corporation).

### Mitophagy assay

Viable cells were stained with 100 nmol/l Mitophagy Dye (Dojindo Laboratories) for 30 min and washed in Hank’s Hepes buffer solution. The attached cells were stimulated with 10 μmol/l carbonyl cyanide m-chlorophenyl hydrazone (Nacalai Tesque) for 24 h before observation, as described in the manufacturer’s protocol. Fluorescent images were obtained using the Leica SP8 X confocal microscope.

### Cellular senescence detection

Frozen sections were fixed with 4% paraformaldehyde for 3 min and incubated with SPiDER-βGal solution (Dojindo Laboratories) at 37 °C for 30 min. After removing the solution, the sections were washed with PBS and mounted with mounting medium including DAPI (Vector Laboratories).

### Isolation of the mitochondrial fraction

Cells were washed in ice-cold PBS and resuspended in subcellular fractionation buffer containing 20 mmol/l Hepes (pH 7.4), 10 mmol/l KCl, 2 mmol/l MgCl_2_, 200 mmol/l sucrose, 1 mmol/l ethylenediaminetetraacetic acid, 1 mmol/l ethyleneglycol tetraacetic acid, 2 mmol/l phenylmethylsulfonyl fluoride (PMSF), and 1 mg/l leupeptin (52). The hearts extracted from mice were washed with PBS, transferred into the subcellular fractionation buffer, and homogenized in the buffer with 15 strokes using the Potter-Elvehjem tissue homogenizer (DWK Life Sciences). Cell and heart samples were then passed through a 26-gauge needle attached to a 1-ml syringe ten times for lysis, followed by centrifugation at 800*g* at 4 °C for 5 min. The supernatant including the mitochondria was transferred into a new tube and centrifuged at 10,000*g* at 4 °C for 5 min. The pellets were resuspended in radioimmunoprecipitation assay buffer containing 50 mmol/l Tris-HCl (pH 7.5), 150 mmol/l NaCl, 0.5% sodium deoxycholate, 0.1% sodium dodecyl sulfate (SDS), 1% Nonidet P-40, 1 mmol/l PMSF, and 1 μg/ml leupeptin to obtain the mitochondrial fraction.

### Western blotting

Mouse and human hearts were homogenized mechanically in radioimmunoprecipitation assay (RIPA) buffer containing 50 mmol/l Tris-HCl (pH7.5), 150 mmol/l NaCl, 0.5% sodium deoxycholate, 0.1% SDS, 1% Nonidet P-40, 1 μg/ml aprotinin, 1 μg/ml leupeptin, 1 mmol/l PMSF, 5 mmol/l NaF, and 1 mmol/l Na_3_VO_4_. HL-1 cells were also lysed in RIPA buffer. The homogenates and lysates were centrifuged at 14,000 rpm for 15 min, and the supernatant was used for further analysis. After the protein concentration in the supernatant was measured by Quick Start Bradford (Bio-Rad Laboratories) using BSA standards, 10 μg of protein samples were separated by 10% or 12% SDS-polyacrylamide gel electrophoresis and transferred to a polyvinylidene difluoride membrane (Bio-Rad Laboratories). The membrane was then blocked for 1 h at room temperature in 5% BSA or 5% skimmed milk in Tris-buffered saline with Tween 20. The membrane was incubated with primary antibody overnight in 5% skimmed milk at 4 °C, followed by incubation with horseradish peroxidase (HRP)-labeled secondary antibody (GE Healthcare) for 1 h in 5% skimmed milk. The membrane was incubated with HRP substrate (Luminata Forte) for 5 min and observed on a luminescent image analyzer (Fusion Solo 6S Edge; Vilber Bio imaging). The band densities were analyzed using ImageJ software.

### Primary antibodies

The detailed information of the primary antibodies used in this study is summarized in Table S1.

### Secondary antibodies

We used the following secondary antibodies: Alexa Fluor 488 goat anti-mouse polyclonal secondary antibody (Cat. No: A-11001, Thermo Fisher Scientific; 1:1000 dilution), Alexa Fluor 488 goat anti-rabbit polyclonal secondary antibody (Cat. No: A-11008, Thermo Fisher Scientific; 1:1000 dilution), Alexa Fluor 555 goat anti-rabbit polyclonal secondary antibody (Cat. No: A27039, Thermo Fisher Scientific; 1:1000 dilution), wheat germ agglutinin conjugated with tetramethylrhodamine (Cat. No: W849, Thermo Fisher Scientific; 5 μg/ml solution), DAPI (Cat. No: NA065, Dojindo Molecular Technologies; 1 μg/ml solution), HRP-linked donkey anti-rabbit IgG secondary antibody (Cat. No: NA934V, GE Healthcare Life Sciences, 1:2000 dilution for Western blotting), and HRP-linked donkey anti-mouse IgG secondary antibody (Cat. No: NA931V, GE Healthcare Life Sciences; 1:2000 dilution for Western blotting).

### ROCK kinase assay

HL-1 cells (6 × 10^5^ cells) were treated with or without 10 μmol/l Y-27632 for 1 h and were lysed in RIPA buffer. After centrifugation at 16,000*g*, the clear supernatant was applied to Cyclex Rho-kinase Assay kit (Medical & Biological Laboratories) for measurements of the kinase activity. The procedures were carried out according to the manufacturer’s instructions, and the optical absorbance was measured at 450 nm with MultiSkan JX (Thermo Fisher Scientific). The background-subtracted values were used for data presentation.

### RNA isolation and quantitative PCR

Total RNA was isolated from human heart samples using TRIzol RNA isolation reagent (Thermo Fisher Scientific). cDNA was synthesized by ReverTra Ace quantitative PCR RT Master Mix with gDNA Remover (Toyobo). After the reverse transcription, quantitative PCR was performed using LightCycler Instrument (Roche Diagnostics). The PCR data were analyzed using standard curve method, and human *GAPDH* mRNA expression level was used as the internal control. The primers for the gene amplifications were as follows: *RHOA* forward (5′-AGCCTGTGGAAGACATGCTT-3′), *RHOA* reverse (5′-TCAAACACTGTGGGCACATAC-3′), *PARK2* forward (5′-CAAGACTCAATGATCGGCAG-3′), *PARK2* reverse (5′- ACACACTCCTCTGCACCATA), *GAPDH* forward (5′-AGCCACATCGCTCAGACAC-3′), *GAPDH* reverse (5′-GCCCAATACGACCAAAATCC-3′).

### AAV serotype 6-mediated Parkin expression

Viral particles containing the AAV serotype 6 vector harboring the *Parkin* and *EGFP* genes linked with the T2A sequence (AAV-Parkin-T2A-GFP) driven by the cytomegalovirus promoter were generated using Vector Builder. The AAV serotype 6 vector carrying only the *EGFP* gene (AAV-GFP) was similarly generated and used as the control. For the recombinant AAVs manufacturing, the plasmid carrying the cDNA of Parkin-T2A-GFP or GFP was transfected into HEK293T packaging cells, together with Rep-cap plasmid and helper plasmid (Vector Builder) encoding adenovirus genes (E4, E2A, and VA) that mediate AAV replication. After a short incubation period, viral particles were harvested from the cell lysate and concentrated by polyethylene glycol precipitation. The viral particles were further purified and concentrated by cesium chloride gradient ultracentrifugation. For measurements of the AAVs titer, digital PCR-based approach was applied. Parkin and GFP expressions in HL-1 cells and mouse hearts were performed by infecting the above viral particles. For the administration of AAV into mice, 1 × 10^11^ viral particles of AAV-Parkin-T2A-GFP or AAV-GFP were intravenously injected through the mouse tail vein after mice were anesthetized with 2% isoflurane. Following AAV serotype 6 injection, HL-1 cells, as well as mouse hearts and lungs, were harvested and isolated, respectively, at the appropriate time points for further analysis.

### Statistical analysis

All numerical values are shown as the mean ± standard deviation. All experiments were performed at least three times independently. Statistical differences between experimental groups were evaluated by two-tailed unpaired Student’s *t* test or one-way or two-way analysis of variance with Bonferroni’s multiple comparison test. The Kaplan–Meier analysis was conducted to evaluate the lifespan of mice in the two groups. The survival curves were compared using the log-rank test. For all analyses, *p* < 0.05 was considered statistically significant.

## Data availability

All of the data are contained within the article and are available from the corresponding author on reasonable request.

## Supporting information

This article contains supporting information.

## Conflict of interest

The authors declare that they have no conflicts of interest with the contents of this article.
