# Supplementary material for: RhoA rescues cardiac senescence by regulating Parkin-mediated mitophagy
Source: J Biol Chem. 2023 Feb 8;299(3):102993. doi: 10.1016/j.jbc.2023.102993 (PMC10020657; doi:10.1016/j.jbc.2023.102993)
Supplement: Supporting Table S1 and Figures S1–S5 [file mmc1.pdf]

Supporting Information

RhoA rescues cardiac senescence by regulating Parkin-mediated mitophagy

Joanne Ern Chi Soh, Akio Shimizu, Md Rasel Molla, Dimitar P. Zankov, Le Kim Chi Nguyen, Mahbubur Rahman Khan, Wondwossen Wale Tesega, Si Chen, Misa Tojo, Yoshito Ito, Akira Sato, Masahito Hitosugi, Shigeru Miyagawa, Hisakazu Ogita

Table S1

Figures S1–S5

Table S1. Information of the primary antibodies used in this study.

Name (Clone No.)	Source	Type	Company	Cat. No.	Dilution ratio
RhoA (67B9)	Rabbit	Monoclonal	Cell Signaling Technology	2117	WB (1:1000), IF (1:200)
p16 (EP1551Y)	Rabbit	Monoclonal	Abcam	ab51243	IF (1:500)
p21 (EPR3993)	Mouse	Monoclonal	Abcam	ab109199	IF (1:500)
GAPDH (3H12)	Mouse	Monoclonal	Medical & Biological Laboratories	M171-3	WB (1:1000)
ATP5A (clone 51)	Mouse	Monoclonal	Santa Cruz Biotechnology	sc-136178	WB (1:1000) IF (1:300)
ATP5A	Rabbit	Polyclonal	Proteintech	14676-1-AP	IF (1:300)
Drp1 (D6C7)	Rabbit	Monoclonal	Cell Signaling Technology	8570	WB (1:1000), IF (1:500)
P-Drp1 [Ser616]	Rabbit	Polyclonal	Cell Signaling Technology	3455	WB (1:1000), IF (1:500)
Parkin (PRK8)	Mouse	Monoclonal	Santa Cruz Biotechnology	sc-32282	WB (1:1000)
Parkin (5A1)	Mouse	Monoclonal	Immuno-Biological Laboratories	10243	IF (1:100)
PINK1(C-3)	Mouse	Monoclonal	Santa Cruz Biotechnology	Sc-518051	WB (1:1000), IF (1:500)
N-Myc (NCM II 100)	Mouse	Monoclonal	Santa Cruz Biotechnology	sc-56729	WB (1:1000), IF (1:300)
P-N-Myc [Ser54]	Rabbit	Polyclonal	Bethyl Laboratories	A300-206A	WB (1:1000), IF (1:300)
Tom20	Mouse	Monoclonal	Santa Cruz Biotechnology	Sc-17764	WB (1:1000) IF (1:300)
Ubiquitin (P4D1)	Mouse	Monoclonal	Santa Cruz Biotechnology	sc-8017	WB (1:1000)
GFP	Rabbit	Polyclonal	Medical & Biological Laboratories	598	WB (1:1000), IF (1:500)

IF: immunofluorescence, WB: western blotting

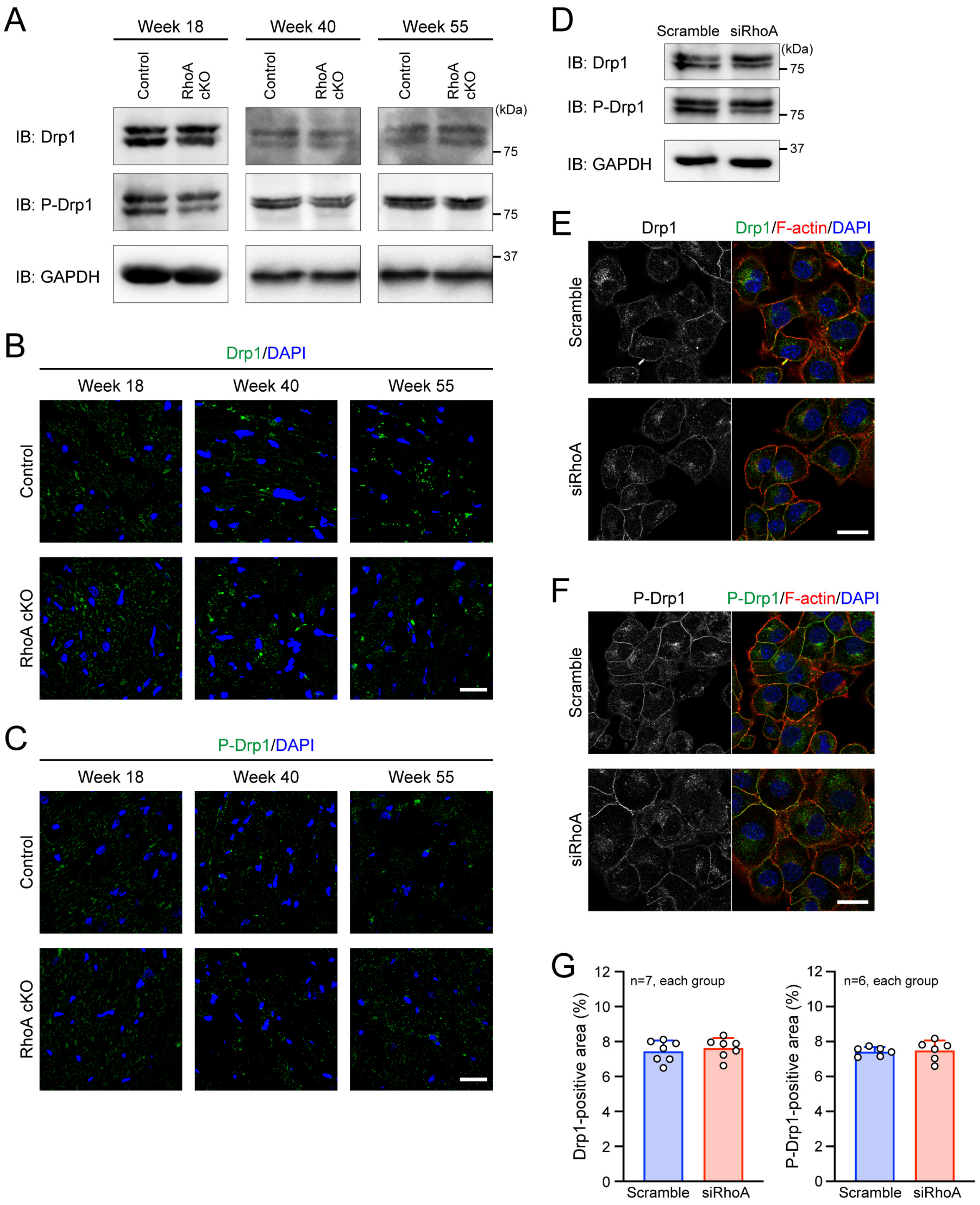

Figure S1. Expression and phosphorylation of the mitochondrial fission protein Drp1 in the mouse heart and cardiomyocytes. (**A**) Western blotting for Drp1 and phosphorylated Drp1 (P-Drp1) in the mouse heart at the indicated time points. (**B** and **C**) Immunostaining for Drp1 (**B**) and P-Drp1 (**C**) in the mouse heart at the indicated time points. (**D**) Western blotting for Drp1 and P-Drp1 in HL-1 cardiomyocytes transfected with siRhoA and scramble RNA as the control. (**E** and **F**) Immunostaining for Drp1 (**E) and P-Drp1** (**F**) in HL-1 cells. (**G**) Summary graphs of the percentage of positive area for Drp1 (**E**) and P-Drp1 (**F**). The data in each graph are shown as the mean ± SD. No difference was observed between groups by *t*-test. GAPDH was blotted as the loading control in (**A** and **D**), and nuclei were counterstained with DAPI in (**B**, **C**, **E**, and **F**). Scale bars: 20 μm.

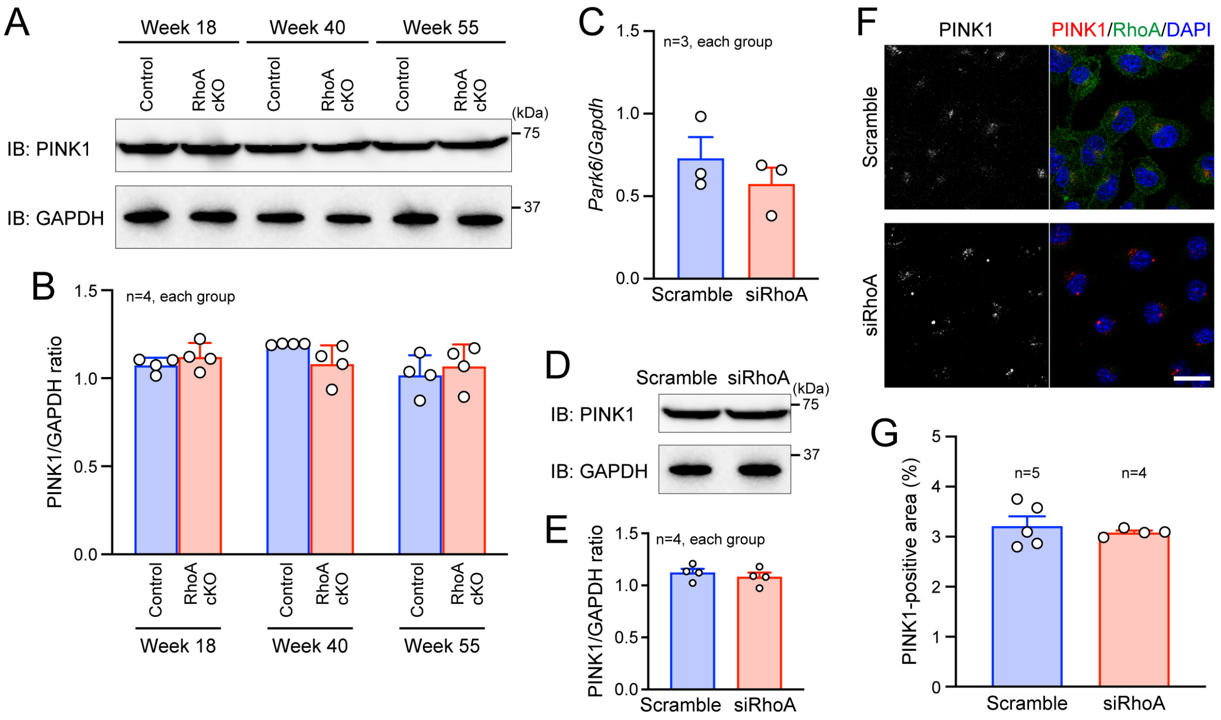

Figure S2. Expression of PINK1 in the mouse heart and cardiomyocytes. (**A**) Western blotting for PINK1 in the mouse heart at the indicated time points. GAPDH was blotted as the loading control. (**B**) Summary graph of the PINK1/GAPDH band ratio. (**C**) qPCR analysis for the gene expression of *Park6*, which encodes PINK1, in HL-1 cardiomyocytes. *GAPDH* gene expression was used as the control. (**D**) Western blotting for PINK1 in HL-1 cells. (**E**) Summary graph of the PINK1/GAPDH band ratio. (**F**) Immunostaining for PINK1 in HL-1 cells. Nuclei were counterstained with DAPI. Scale bar: 20 μm. (**G**) Summary graph of the percentage of PINK1-positive area. The data in each graph are shown as the mean ± SD. In (**B**, **C**, **E**, and **G**), *t*-test was used to compare the data between groups.

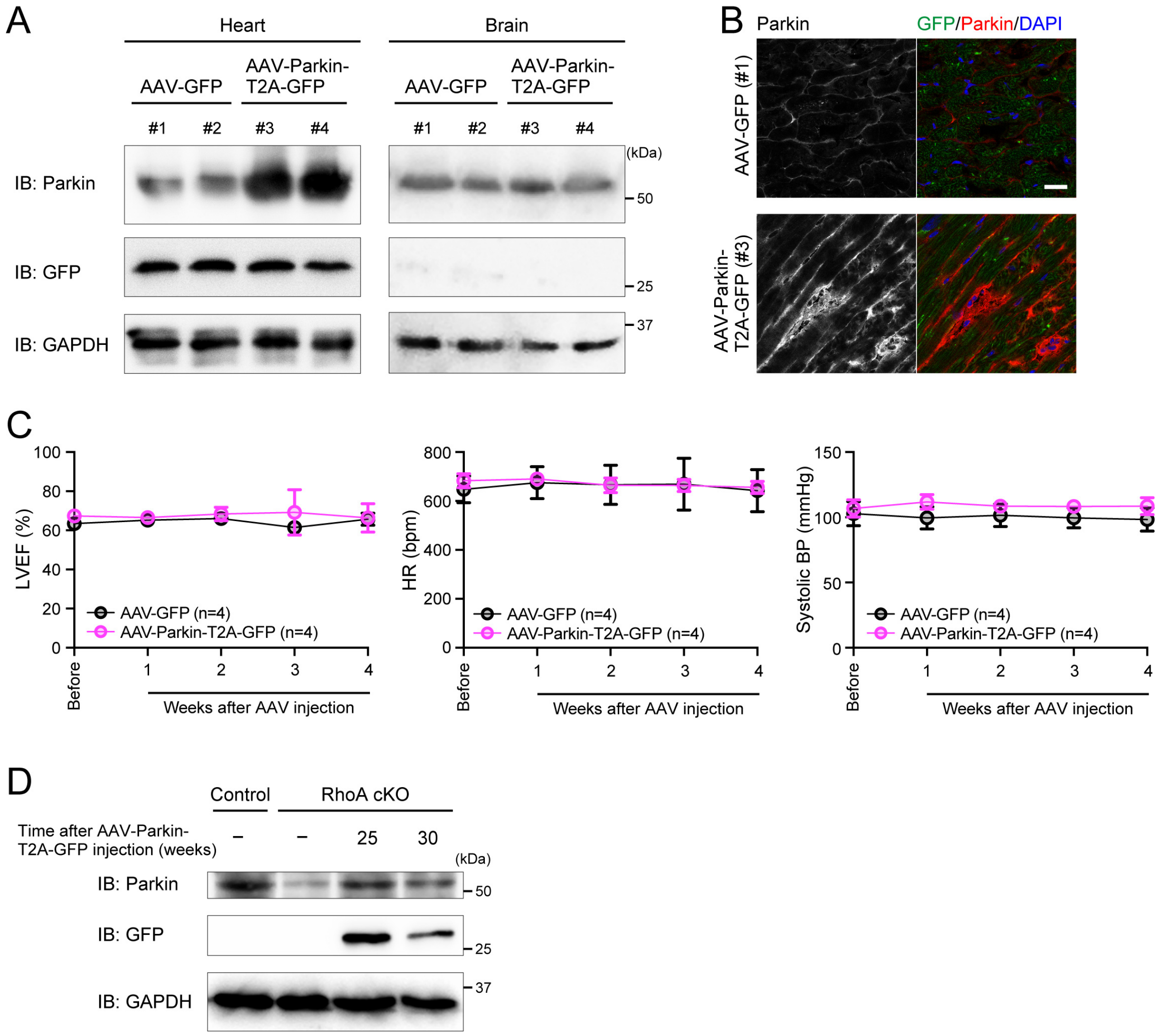

Figure S3. Expression of Parkin in the mouse heart after intravenous injection of AAV-Parkin. (**A**) Western blotting for Parkin and GFP in the mouse heart and brain 4 weeks after injection of AAV-Parkin-T2A-GFP or AAV-GFP through the tail vein in control mice. GAPDH was blotted as the loading control. (**B**) Immunostaining for Parkin in the control mouse hearts 4 weeks after AAV-Parkin-T2A-GFP or AAV-GFP injection. Nuclei were counterstained with DAPI. Scale bar: 20 μm. (**C**) No changes were observed in cardiac function or hemodynamics in control mice after AAV-Parkin-T2A-GFP or AAV-GFP injection. The data in each graph are shown as the mean ± SD. (**D**) Western blotting for Parkin and GFP in the mouse heart to examine the persistence of Parkin expression after AAV-Parkin-T2A-GFP injection.

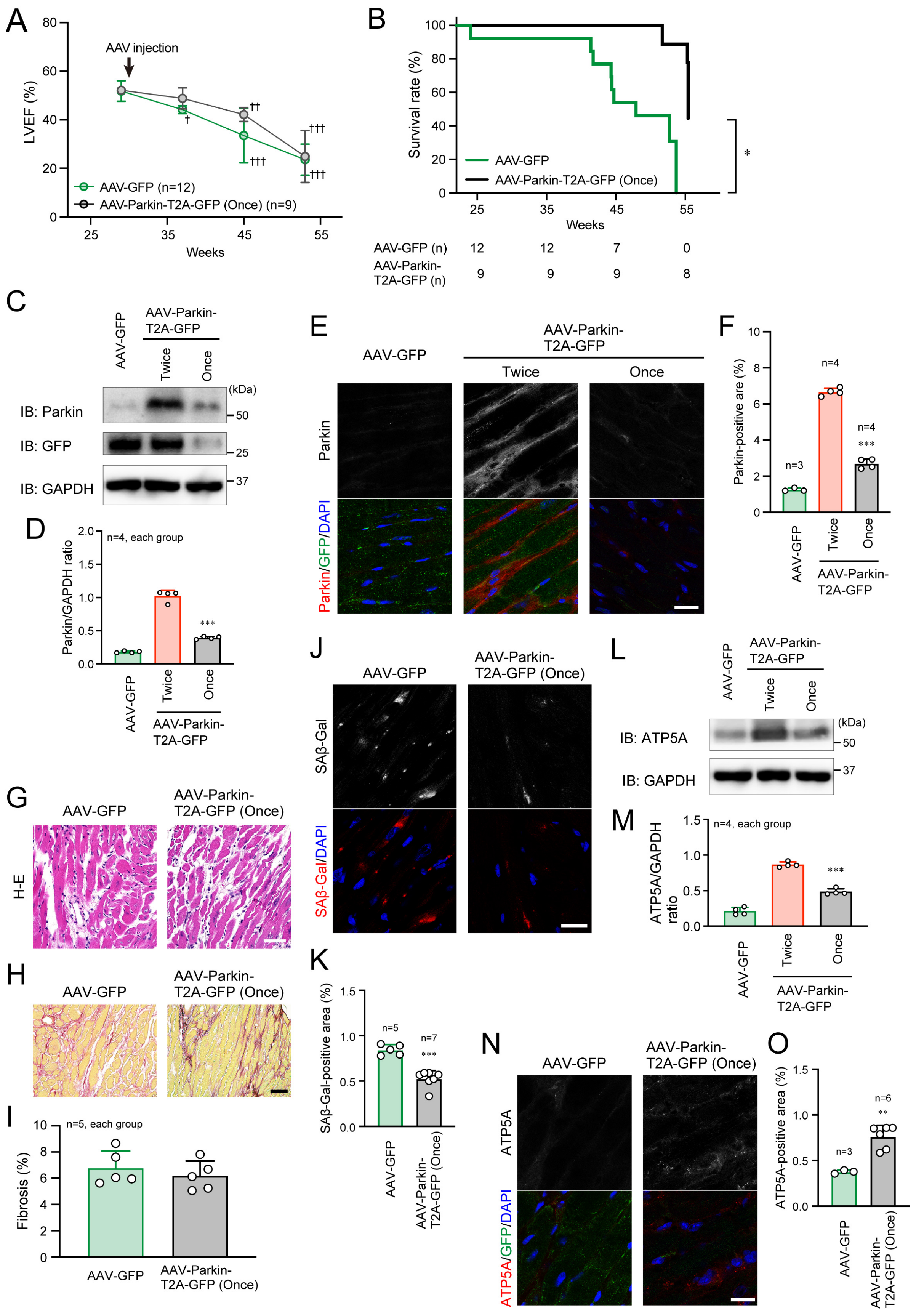

Figure S4. Late administration of AAV-Parkin in RhoA cKO mice. (**A**) LVEF analyzed by echocardiography in 29-, 37-, 45-, and 53-week-old mice. AAV-Parkin-T2A-GFP or AAV-GFP as

the control was intravenously injected into RhoA cKO mice once at 30 weeks after birth. (**B**) Kaplan–Meier survival curve of RhoA cKO mice after AAV-Parkin-T2A-GFP or AAV-GFP injection. The number of mice (n) in each group at every 10 weeks is indicated below the graph. (**C**) Western blotting for Parkin and GFP in the mouse hearts from 53-week-old mice. (**D**) Summary graph of the Parkin/GAPDH band ratio. (**E**) Immunostaining for Parkin in the 53-week-old mouse heart. Scale bar: 20 μm. (**F**) Summary graph of the percentage of Parkin-positive area. (**G** and **H**) H-E staining and Picro-sirius red staining of the 53-week-old mouse hearts. Scale bars: 50 μm. (**I**) Summary graph of the percentage of cardiac fibrosis examined in (**H**). (**J**) Immunostaining for the senescence marker SAβ-Gal in the 53-week-old mouse heart. Scale bar: 20 μm. (**K**) Summary graph of the percentage of SAβ-Gal-positive area. (**L** and **N**)Western blotting (**L**) and immunostaining (**N**) for ATP5A in the 53-week-old mouse heart. Scale bar: 20 μm. (**M** and **O**) Summary graphs of the ATP5A/GAPDH ratio (**M**) and the percentage of ATP5A-positive area (**O**) examined in (**L** and **N**), respectively. GAPDH was blotted as the loading control in (**C** and **L**), and nuclei were counterstained with DAPI in (**E**, **J**, and **N**). The data in each graph are shown as the mean ± SD. In **A**, two-way ANOVA and one-way ANOVA were used to compare the data between groups and weeks, respectively, and in (**B**), the data were analyzed by Kaplan–Meier method. One-way ANOVA (**D**, **F**, and **M**) or *t*-test (**I**, **K**, and **O**) was used to compare the data between groups. * $p < 0.05$, ** $p < 0.01$, and *** $p < 0.001$ vs. AAV-GFP; † $p < 0.05$, †† $p < 0.01$, and ††† $p < 0.001$ vs. Week 29.

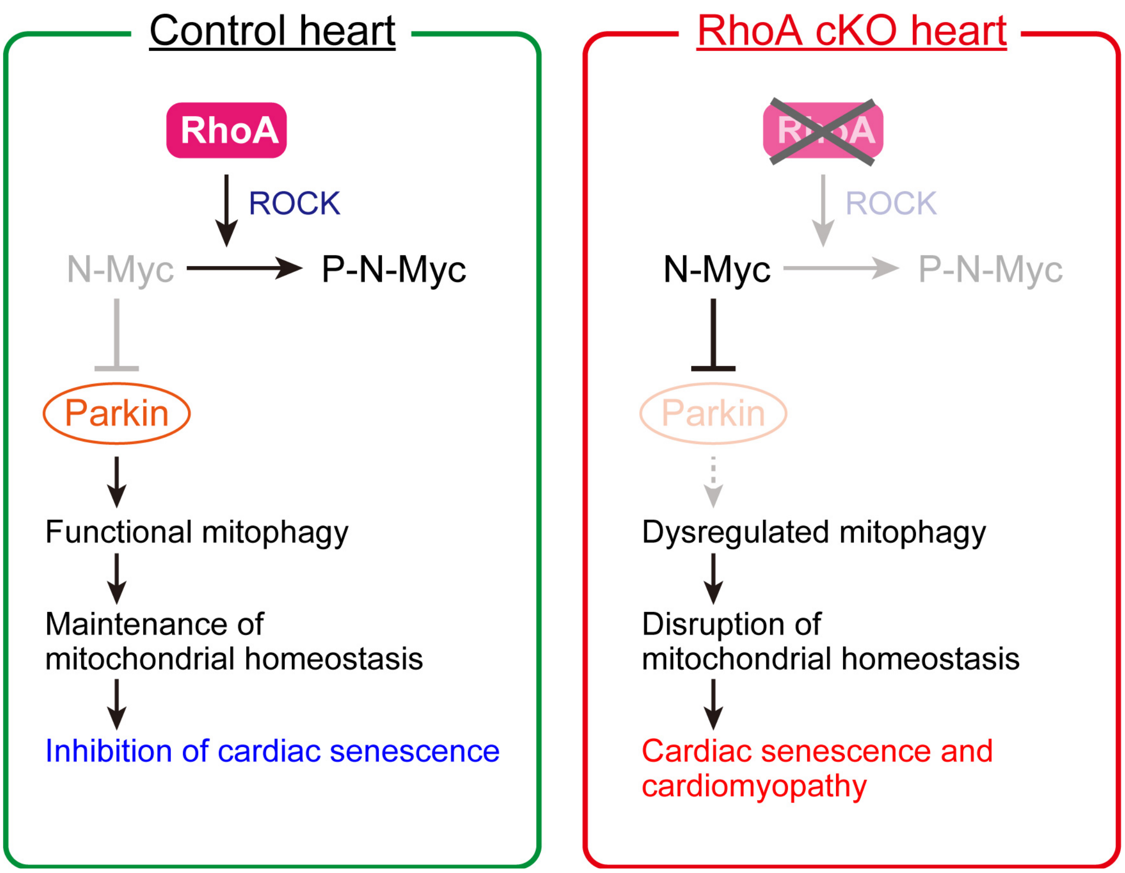

Figure S5. Schematic representation of the function of RhoA in the heart. RhoA up-regulates the expression of Parkin in cardiomyocytes to maintain mitochondrial homeostasis, resulting in inhibition of cardiac senescence and heart failure.
